# Cardiotoxicity of Cancer Therapeutics: Current Issues in Screening, Prevention, and Therapy

**DOI:** 10.3389/fphar.2013.00019

**Published:** 2013-03-12

**Authors:** Richard J. Sheppard, Jenna Berger, Igal A. Sebag

**Affiliations:** ^1^Division of Cardiology, Sir Mortimer B. Davis-Jewish General Hospital and Lady Davis Institute for Medical ResearchMontreal, QC, Canada; ^2^Faculty of Medicine, McGill UniversityMontreal, QC, Canada

**Keywords:** chemotherapy, cardiotoxicity, predictors, anthracyclines, trastuzumab, heart failure, echocardiography

## Abstract

In the context of modern cancer chemotherapeutics, cancer survivors are living longer and being exposed to potential comorbidities related to non-cancer side effects of such treatments. With close monitoring of cancer patients receiving potentially cardiotoxic medical therapies, oncologists, and cardiologists alike are identifying patients in both clinical and subclinical phases of cardiovascular disease related to such chemotherapies. Specifically, cardiotoxicity at the level of the myocardium and potential for the development of heart failure are becoming a growing concern with increasing survival of cancer patients. Traditional chemotherapeutic agents used commonly in the treatment of breast cancer and hematologic malignancies, such as anthracyclines and HER-2 antagonists, are well known to be associated with cardiovascular sequelae. Patients often present without symptoms and an abnormal cardiac imaging study performed as part of routine evaluation of patients receiving cardiotoxic therapies. Additionally, patients can present with signs and symptoms of cardiovascular disease months to years after receiving the chemotherapies. As the understanding of the physiology underlying the various cancers has grown, therapies have been developed that target specific molecules that represent key aspects of physiologic pathways responsible for cancer growth. Inhibition of these pathways, such as those involving tyrosine kinases, has lead to the potential for cardiotoxicity as well. In view of the potential cardiotoxicity of specific chemotherapies, there is a growing interest in identifying patients who are at risk of cardiotoxicity prior to becoming symptomatic or developing cardiotoxicity that may limit the use of potentially life-saving chemotherapy agents. Serological markers and novel cardiac imaging techniques have become the source of many investigations with the goal of screening patients for pre-clinical cardiotoxicity. Additionally, studies have been performed.

## Introduction

Improving survival in patients with cancer is related to early diagnosis and novel, increasingly effective therapies. This has consequently exposed patients to increasingly more frequent cardiovascular (CV) morbidities and mortalities related to established risk factors. In addition, the chemotherapy (CT) itself has become a “risk factor” for CV disease in patients with cancer. The CV side effects of these chemotherapeutic agents are becoming increasingly apparent and prevalent based on close clinical monitoring, the use of serological markers, such as troponins and BNP, and finally by using standard and novel imaging techniques, which includes nuclear imaging, echocardiograms, and MRI.

Cardiologists are taking on a more prominent role in the care of patients with cardiotoxicity from agents used to treat breast cancer as well as hematological and other malignancies. Early identification of patients who are at risk for chemotherapy-induced cardiotoxicity (CIC) should be a primary goal in the development of individualized therapeutic strategies and interventions in cancer patients (Albini et al., 2010). The therapeutic options have been studied in only a handful of trials, including the traditional heart failure (HF) medications such ACE inhibitors, angiotensin receptor antagonists (ARA), and beta blockers. Traditional chemotherapeutic agents, such as anthracyclines (AC) have been known to cause CV sequelae for many years, however now with the development of more targeted drugs such as monoclonal antibodies and tyrosine kinase inhibitors (TKI), new CV side effects are becoming more prevalent. The topics of CT and cardiotoxicity, its therapies, prevention, and potential predictors will be reviewed.

## Chemotherapeutic Agents

There is an extensive list of chemotherapeutic agents that have the potential to induce HF or predispose patients to cardiotoxicity of the myocardium, including anthracycline agents, certain hormonal therapies like Trastuzumab, TKI, 5 Fluorouracil and its related compounds, and several others. The focus of this review is on the therapies that have been recently evaluated in the context of their impact on not only clinical HF presentation but on cardiac biomarkers and imaging techniques.

### Anthracyclines

Anthracyclines are one of the most widespread chemotherapeutic agents often used for the treatment of lymphomas, hematological malignancies, breast carcinomas, and sarcomas. They are a class of drugs derived from the *Streptomyces* bacterium and prevent DNA transcription and replication and potentially generate free radicals that can damage the DNA (Chaires, 1990).

The true incidence of chronic cardiotoxicity related to AC has been difficult to determine accurately because the follow-up duration has been brief in most clinical trials. Moreover, studies have variable definitions of cardiotoxicity and different methods used for measurement of cardiac function (Barrett-Lee et al., 2009). Initially, early studies demonstrated that the incidence of HF was approximately 3.0% in patients receiving a cumulative dose of doxorubicin of 400 mg/m^2^, 7.5% at 550 mg/m^2^, and 18.0% at 700 mg/m^2^ (Von Hoff et al., 1979). In a retrospective study done years later, the incidence was found to be 5.0% at a cumulative dose of 400 mg/m^2^, 26.0% at 550 mg/m^2^, and 48.0% at 700 mg/m^2^ and it was hypothesized that AC-induced cardiotoxicity had been previously under estimated (Swain et al., 2003). More than one half of all patients exposed to AC will present with some degree of cardiac dysfunction 10–20 years after CT (Steinhertz et al., 1991) and AC-induced cardiomyopathy has been associated with a 2-years mortality rate up to 60% (Cardinale et al., 2010).

Anthracycline’s are associated with an irreversible form of dilated cardiomyopathy and dose dependant cardiotoxicity (Yeh et al., 2004). They have revolutionized the management of certain malignancies, but there remains a clinically significant incidence of cardiotoxicity associated with these drugs. AC-induced cardiotoxicity may be acute, subacute, or chronic. Acute or subacute cardiotoxicity is rare, usually independent of dose, and reversible. It may present as asymptomatic electrocardiographic changes, arrhythmias, heart block, or more rarely an acute myocarditis. It can potentially resolve after discontinuation of the therapy. Chronic or late-onset cardiotoxicity may present months or years after infusion and tends to be an irreversible cardiomyopathy (Barrett-Lee et al., 2009). The onset of AC-induced cardiac dysfunction, even asymptomatic may negatively affects cancer patient’s cardiac outcomes and limit their therapeutic opportunities (Cardinale et al., 2006).

### Monoclonal antibody

#### Trastuzumab (herceptin)

Trastuzumab is a humanized monoclonal antibody aimed at targeting ERB2 (epidermal growth factor receptor 2) on the surface of ERB2 overexpressing tumor cells. Trastuzumab is approved for the treatment of ERB2 positive breast cancer in both the metastatic and adjuvant settings (Carver, 2010). Trastuzumab associated cardiac dysfunction was first identified in metastatic breast cancer trials and second to AC, it is the most common chemotherapeutic agent associated with left ventricular dysfunction (Witteles et al., 2011).

Slamon et al. (2001) were amongst the first to identify trastuzumab-related cardiotoxicity. Women with progressive metastatic breast cancer, with overexpression of HER-2, who had not previously received CT for metastatic disease, were included in this study. Patients were randomly assigned to receive standard CT alone or CT plus trastuzumab. The most noted adverse event was cardiac dysfunction, which occurred in 27% of those who received AC, cyclophosphamide, and trastuzumab; 13% of those given paclitaxel and trastuzumab and 1% of those given paclitaxel alone. The incidence of NYHA 3/4 or 4/4 HF was highest among patients who had received an AC, cyclophosphamide, and trastuzumab.

Unlike the cardiac dysfunction caused by AC, that associated with trastuzumab is unrelated to dose and reversible in many cases. Thirty eight patients with HER-2/neu-positive breast cancer were referred for suspected trastuzumab-related cardiotoxicity and were studied over a 4-years period (Ewer et al., 2005). After a median of 4.5 months of trastuzumab therapy, the mean LVEF decreased below baseline. Once left ventricular dysfunction or HF was identified, trastuzumab was discontinued. The patients were either treated for cardiac dysfunction or observed. During a period of approximately 1.5 months, the LVEF returned toward baseline and at 1–3 months, improved in all of the patients except the one patient who continued on trastuzumab for palliative reasons. Nine patients underwent right ventricular endomyocardial biopsy, which did not demonstrate any evidence of typical AC-related changes. Further studies are warranted in order to investigate the ideal timing between AC and trastuzumab therapy in order to minimize cardiac damage.

#### Tyrosine kinase inhibitors

Tyrosine kinase inhibitor’s are molecules that were designed to target TKs which are overexpressed in cancer cells. However, they may also inhibit normal aspects of TK functions in non-cancerous cells and may lead to undesirable side effects such as cardiotoxicity and left ventricular failure (Chu et al., 2007). They have led to a tremendous advancement in the treatment of malignancies such as breast cancer, renal cell carcinoma (RCC), lymphoma, and gastrointestinal stromal tumors (GIST).

#### Imatinib (gleevec)

Imatinib is a TKI, of the ABL kinase, which has been approved and effective for the treatment of malignancies such as chronic myeloid leukemia (CML) and GIST (Garcia-Alvarez et al., 2010). Cardiotoxicity associated with Imatinib has not been well established. Early clinical trials investigating the utility of Imatinib in GIST treatment had not consistently monitored cardiac function.

In the International randomized Study of Interferon versus STI571 (IRIS) study there was no clinical or statistical difference in the incidence of HF between the imatinib and interferon arms (O’Brien et al., 2003). Clinical data from 10 patients, who developed left ventricular dysfunction after treatment with Imatinib, was collected (Kerkela et al., 2006). LVEF had been normal prior to therapy but dropped to an average of 25% after a mean of 7.2 months. However, it was concluded with later studies that this mainly affected elderly individuals with pre-existing comorbidities.

Due to the concern of the potential cardiac damage induced by this medication, the European Organization for Research on Treatment Cancer-Italian Sarcoma Group-Australian Gastrointestinal Trials Group (EORTC-ISG-AGITG) database was explored and 946 GIST patients were studied for cardiac damage secondary to Imatinib therapy (Verweij et al., 2007). Cardiac assessment was based on the patient’s history and physical exam. Overall, 10 patients were reported to have had a cardiac event or reported HF. However, when analyzed in more detail, two patients had previous HF due to previous AC exposure and two other patients had known LV dysfunction, and most patients continued imatinib without any further cardiac complications, concluding that HF directly secondary to Imatinib was less likely.

Upon further investigation, 1,276 patients with CML who had been enrolled in clinical trials of imatinib from 1998 to 2006 were investigated for cardiac events secondary to Imatinib (Atallah et al., 2007). Among these patients, 22 (1.8%) were reported as having symptoms that could be attributed to HF, with 18 of these patients having previous medical conditions predisposing them to cardiac disease and 13 patients having previously known cytotoxic drugs. The authors concluded that HF caused directly by imatinib is rare and it was not possible at the time to establish a direct link between the two due to the multiple cofounders.

#### Sunitinib (sutent)

Sunitinib is a multi-targeted TKI that prolongs survival in patients with GIST and RCC. It targets vascular endothelial cell growth factor receptors (VEGFR)1–3, platelet derived growth factor receptors (PDGRF) alpha and beta, FMS-like tyrosine kinase-3 (Flt-3), c-kit (stem cell factor receptor), colony stimulating factor 1 receptor (CSF1R), and the ret oncogene product (Chu et al., 2007). The CV risk associated with sunitinib in patients with metastatic GIST was evaluated in a retrospective study (Chu et al., 2007). All CV events in 75 patients with imatinib-resistant, metastatic, GIST had been enrolled in a phase I/II trial investigating the efficacy of sunitinib. After the fourth cycle, more than 50% of patients were started on beta blockers or ACEI for hypertension control. All had a baseline LVEF of >50% and none had a history of HF. Of the 75 patients, 36 went off secondary to disease progression. Three of 36 (8%) patients treated with sunitinib developed NYHA class 3–4 HF.

Motzer et al. (2006) reported that 10% of subjects in their clinical trial experienced a drop in LVEF. However, this drop in LVEF was not associated with clinical symptoms and was reversible after modification of the dose or discontinuation of treatment. Due to conflicting data and the lack of knowledge of the true risk of HF with sunitinib, a meta-analysis was carried out in order to determine the incidence and risk of developing HF in patients receiving sunitinib therapy (Richards et al., 2011). Only phase II and III studies were included and patients with significant CV disease, dysrhythmias, prolongation of the QT interval, or uncontrolled hypertension were excluded. Sixteen total studies with 6,935 patients were included as well as both randomized and non-randomized studies. HF regardless of grade represented a total incidence of 4.1% whereas high-grade HF represented an incidence of 1.5%. An overall RR of developing all-grade HF was 1.81 and 3.3 for high-grade HF events.

#### Bevacizumab (avastin)

Vascular endothelial growth factor (VEGF) plays a role in tumor growth, invasion, and metastasis by promoting tumor angiogenesis (Ranpura et al., 2010) and in knock-out mice, lacking the VEGF gene, the myocardial walls were thinned and contractile function was decreased (Giordano et al., 2001). Bevacizumab is a humanized monoclonal antibody that inhibits the activity of VEGF and has been shown to be beneficial in the treatment of metastatic colorectal cancer, non-small cell lung cancer, and breast cancer (Izzedine et al., 2009). A meta-analysis was conducted studying the risk of HF in breast cancer patients receiving bevacizumab in randomized trials (Choueiri et al., 2011). This is of importance since many breast cancer patients have had prior exposure to cardiotoxic agents such as AC and/or trastuzumab. The overall incidence of high-grade HF was 1.6%. For the control group or placebo-treated group, there was an incidence of 0.4%. The overall RR of developing high-grade HF with bevacizumab was 4.74. When directly comparing the incidence and RR of HF with high- versus low-dose bevacizumab, no significant difference was found. The concomitant CTs administered were taxanes, capecitabine, or ACs and there were no significant difference between the different CTs and the incidence of HF.

The use of TKIs has drastically improved the survival and prognosis of various malignancies. However, these molecules also play a role in the function of cardiac cells and may impede on their function. In addition to the potential for hypertension as a related CV consequence of TKI use, patients with previous cardiac risk factors are at increased risk for TKI induced cardiotoxicity, therefore these must be assessed prior to the beginning of treatment.

## Cardiovascular Therapies for Chemotherapy Related Cardiotoxicity

Anthracycline-induced cardiotoxicity has been well established for many years and only a handful of prospective studies have been published, exploring therapeutic options to treat or prevent CIC (Table 1). Most data are based on findings reported in older studies (Cardinale et al., 2010). Trastuzumab associated toxicity usually responds to standard HF treatment or the discontinuation of trastuzumab, although recovery rates may be lower in those who have received prior ACs. The majority of trastuzumab-related cardiac events are asymptomatic declines in LVEF and early detection and management is crucial to recovery. There is very limited data on specific CV therapeutics in the management of TKI induced cardiotoxicity.

**Table 1:** **Clinical studies of medical therapy in chemotherapy-induced cardiotoxicity**.

Reference	No of participants	Therapy	Significant clinical endpoints	Follow-up duration
Nakame et al. (2005)	40	Valsartan 80 mg daily vs. control	BNP (pg/ml)	7 days
			LVDD (mm)	
			QTc (ms)	
Ewer et al. (2005)	38	Removal of trastuzumab ± ACEI and beta blockers (tolerated dose)	LVEF (%)	4.5 months (median)
Cardinale et al. (2006)	114	Enalapril 2.5–20 mg daily vs. control	LVEF (%)	12 months
			EDV (ml)	
			ESV (ml)	
Kalay et al. (2006)	50	Carvedilol 12.5 mg daily vs. control	LVEF (%) E/A ratio	6 months
Cardinale et al. (2010)	201	Enalapril ± carvedilol (tolerated dose)	LVEF (%) time to HF treatment (months) NYHA functional class	36 ± 27 months
Acar et al. (2011)	40	Atorvastatin 40 mg daily vs. control	LVEF (%)	6 months
			LVEDD (mm)	
			LVESD (mm)	

*LVEF, left ventricular ejection fraction*.

*BNP, brain natriuretic peptide*.

*ESV, end-systolic volume*.

*LVESD, left ventricular end-systolic diameter*.

*LVDD, left ventricular end-diastolic diameter*.

*EDV, end-diastolic volume*.

*LVEDD, left ventricular end-diastolic diameter*.

In 2005, Nakame et al. (2005) aimed to characterize acute CHOP (cyclophosphamide, doxorubicin, Vincristine, and prednisolone) induced cardiotoxicity using echocardiography, ECG as well as serum cardiac markers in order to evaluate whether or not Angiotensin II type 1 receptor blockers (ARBs) can prevent acute CHOP induced cardiotoxicity in patients with Non-Hodgkins Lymphoma (NHL). None of the patients had received prior CT or radiation therapy and all had an LVEF >50% at baseline. Forty patients with untreated NHL who were scheduled to undergo CHOP therapy were randomized to receive 80 mg daily of Valsartan vs. placebo, from the first day of CHOP therapy. The doxorubicin dose in the CT regime was 50 mg/m^2^. An echocardiogram was performed at baseline, 3, 5, and 7 days after initiation of CHOP. It was demonstrated that patients not treated with Valsartan had an increased LVDd (end-diastolic diameter), BNP, ANP, QTc interval. There were no significant differences with LV end-systolic diameter, LVEF, E/A, and DcT. There was some impact on some specific CV parameters; Valsartan however, significantly inhibited the dilatation of LVDd, elevation of BNP, and prolongation of the QTc interval and QTc dispersion.

In 2005, Ewer et al. (2005) studied trastuzumab-related cardiotoxicity. Of the 37 patients affected, 15 of these patients experienced symptomatic HF. They were all treated with standard HF medications, including ACEI and beta-blockers. All of the 20 patients with HF had some improvement in their LVEF and significant symptomatic improvement when trastuzumab was discontinued; however, two of the five patients who were not treated with HF medications had persistent LV dysfunction when reassessed approximately 6 months later. Once the LVEF stabilized and HF symptoms improved, 25 of the 38 patients were reintroduced to trastuzumab because they had a positive initial response. Among those who restarted treatment, 22 of the 25 patients had stable LVEF measurements once trastuzumab was reinitiated, without recurrences of HF during follow-up. LV dysfunction and/or HF reoccurred in the remaining three patients and trastuzumab therapy was permanently discontinued. Therefore, patients who experienced any form of cardiotoxicity while receiving trastuzumab therapy generally recovered when it was discontinued and this improvement occurred over a period of months (mean time, 1.5 months).

A prospective, randomized clinical study evaluated the effect of enalapril treatment on the prevention of cardiotoxicity in cancer patients undergoing high dose chemotherapy (HDC) that is followed by an increase in TnI (Cardinale et al., 2006). Among 473 patients undergoing HDC, 114 showed increased TnI values soon after HDC and participated in the study. All patients with an early TnI value >0.07 ng/ml received enalapril vs. placebo. In the ACEI group, enalapril was started 1 month after the end of the last cycle of HDC and was continued for 1 year. Enalapril was started at a dose of 2.5 mg daily and gradually increased to 20 mg once daily. Throughout the study, a progressive decline in LVEF and an increase in end-diastolic and end-systolic volumes were found only in the control subjects. Thirty one cardiac events occurred during follow-up with the incidence highest in the control subjects. Specifically, 14 events of “HF” occurred in the control group and none in the ACEI group. Early treatment with an ACEI, once myocardial damage becomes apparent (rise in TnI), was postulated to prevent the development of cardiotoxicity.

The use of carvedilol in preventing AC-induced cardiomyopathy was evaluated in a single blind, placebo controlled study (Kalay et al., 2006). The carvedilol group received 12.5 mg daily and started prior to CT and maintained treatment for 6 months during CT. None of the subjects received any prior CT or radiation therapy. There was a greater drop in LVEF in patients who did not receive beta blockers. In addition, E/A ratios were significantly reduced on doppler studies in the control group, however, the mortality difference between the two groups was not significant. Of note is that carvedilol was the chosen agent since it has been demonstrated to possess superior antioxidant and anti-apoptotic properties in previous studies.

In 2010, Cardinale et al. (2010) evaluated a large population of symptomatic and asymptomatic patients with AC-induced cardiomyopathy and examined their response to current HF medical therapy. This prospective study included patients with evidence of left ventricular systolic dysfunction (LVEF < 45%) secondary to AC use. The primary end point was LVEF response to HF therapy. Therapy with enalapril and subsequently carvedilol was initiated after detection of LVEF impairment. In 201 patients, 72 received only enalapril and 129 received both enalapril and carvedilol. Time to HF treatment and NYHA functional class were selected as the only independent predictors of lack of complete LVEF recovery. When patients with both a time to HF treatment <6 months and an NYHA class 1 or 2 were considered, positive predictive value for complete LVEF recovery was 84% and negative predictive value was 87%. If a time to HF treatment was greater than 6 months duration or NYHA 3/4, no patient experienced LVEF recovery. As observed in this study, baseline NYHA functional class 3 or 4 is a strong predictor of lack of response to HF therapy.

The use of statins has been accepted as common practice to prevent and treat atherosclerotic disease due to their lipid lowering and anti-inflammatory effect. However, they also possess an anti-oxidative effect which may be beneficial in preventing CIC. A prospective study of 40 patients was performed in order to determine if the use of atorvastatin would prevent a decrease in LVEF post AC CT (Acar et al., 2011). Patients were randomized into a statin vs. control group. The statin group received 40 mg/day of atorvastatin before CT and continued on this medication for 6 months afterward. On control echocardiography, 6 months post CT, there was no significant difference in LVEF in the statin group however there was a statistically significant drop in EF in the control group. Additionally, in the control group, there was a significant increase in LVEDD and LVESD.

A retrospective study evaluated the use of continuous statin treatment on the development of HF in women with breast cancer, who had undergone AC based CT (Seicean et al., 2012). The primary outcome was new onset HF causing hospitalization. Study subjects were found on electronic medical records and women with breast cancer on continuous statin treatment were matched with controls. Based on observational data, patients who were on continuous statin treatment were more likely to be older and have hypertension and therefore be on concurrent ACEI and BB therapy. The incidence of HF was found to be lower in the statin group; specifically there were four cases of HF in the statin group compared to 23 cases in the controls.

The lack of long-term studies has made it difficult to establish a set of guidelines to assist us in the management of CIC. Further prospective studies, investigating the ideal timing of introduction of medical therapy and the choice of therapies are warranted in order to preserve cardiac function.

### Early predictors of chemotherapy-induced cardiotoxicity

As potential CV and imaging screening tools in order to detect the presence of subclinical CV disease, biomarkers may be useful in identifying patients at an elevated risk of disease. In addition, they may be helpful as prognostic indicators in those who have already developed CV sequelae and would be useful in guiding us in concurrent management (Ky and Carver, 2011). Several biomarkers and imaging parameters have been examined in the context of CIC in order identify or stratify risk in those patients that would potentially benefit from early cardioprotective measures (Ky and Carver, 2011).

### Serological markers

#### Troponins

Cardiac troponins I and T are cardiac regulatory proteins that control the calcium-mediated interaction of actin and myosin. Cardiac troponin I is a marker of minor myocardial damage and it is released by cardiac cells in proportion to the degree of myocardial damage (Cardinale et al., 2000). Cardiac troponin I has not been found outside the cardiac muscle, as opposed to cardiac troponin T where it is mildly expressed in skeletal muscle. However, with the second generation assays, cardiac troponin T should only be detected if it is arising rise cardiac tissue (Ricchiuti et al., 1998).

Two large studies were carried out in order to investigate the value of Troponin I as an indicator of early myocardial damage and as a predictor of future CV events in CIC. Following high dose CT, troponin I measurements were used as a predictor of the resulting CV damage (Cardinale et al., 2000). Two hundred and four patients, previously treated with ACs or radiotherapy were assigned to two groups. The cTnI+ group was defined by a value ≥0.5 ng/ml, while the cTnI-group was defined by a value <0.5 ng/ml. Patients in whom TnI were negative, on follow-up echocardiography, tended to have a transient decrease in LVEF after 3 months but recovered to an LVEF greater than 50% by 4–7 months. Among patients in whom TnI were positive, the drop in LVEF was more substantial from 3 months onward, continued to drop and maintained at a decreased level until the end of follow-up. The percentage of cTnI positivity increased in parallel with the increasing number of the cycles performed.

The same authors set out to examine the potential impact of the rise in TnI beyond the early rise in troponins (Cardinale et al., 2004). A total of 703 patients were enrolled. Early troponins (E-TnI) consisted of samples taken within 3 days after CT infusion whereas late troponins (L-TnI) were drawn 1 month post CT. Patients without TnI elevation after HDC had a good prognosis; no significant reduction in LVEF was observed in this group, and a very low incidence of cardiac events (1%) occurred during the follow-up. The PPV was 84% and NPV 99%. The high NPV allows the identification of low-risk patients who may not require very close cardiac surveillance following HDC.

Recently new generation highly sensitive Troponin T (hsTnT) assay demonstrated prognostic value in a stable HF population (Dodos et al., 2008). Forty three women were studied prior to, in addition to 3 and 6 months post CT in order to assess whether early biomarkers could predict the development of CIC in patients treated with ACs and trastuzumab (Sawaya et al., 2011). Elevation of high sensitivity troponin I cardiac markers at 3 months post CT was able to detect a decrease in LVEF in six of nine patients, therefore the authors concluded that this was an independent risk factor for the development of cardiotoxicity at 6 months post CT.

#### Natriuretic peptides

Natriuretic peptides (ANP, BNP, and NT-proBNP) are produced by the myocardium in response to wall strain and pressure overload. They have been recognized and incorporated in guidelines for diagnosis and management of HF (Horacek et al., 2007). Plasma levels of natriuretic peptides have been shown to be increased in patients with severe HF but also in those patients with asymptomatic left ventricular dysfunction (Nousiainen et al., 1999). Serum concentrations of natriuretic peptides have also been measured in patients undergoing CT in order to try and predict the risk of CV damage, but results have been less consistent than the research with troponins. The few studies evaluating their use will be presented.

Thirty adult patients with previously untreated non-Hodgkin’s lymphoma, who were scheduled to receive CHOP, were prospectively studied in order to determine whether these natriuretic peptides could be used to predict impairment of left ventricular function (Nousiainen et al., 1999). LVEF was measured with radionuclide ventriculography (RVG) and it was performed at baseline and after the cumulative doxorubicin doses of 200, 400, and 500 mg/m^2^. Natriuretic peptide measurements were taken at baseline, before every treatment course and 4 weeks after the last CHOP. ANP levels increased steadily after the doxorubicin cumulative dose of 200 mg/m^2^ and reached significant levels at the cumulative dose of 500 mg/m^2^. A trend was detected between an increase in plasma NT-proANP and decrease in LVEF after the cumulative doxorubicin dose of 500 mg/m^2^. Finally, there was a trend between the increase in plasma BNP and the decrease in LVEF after the cumulative doxorubicin dose of 400 mg/m^2^. This study focused on systolic cardiac function and did not correlate the natriuretic factors with any diastolic parameters.

The possible predictive role of NT-proBNP was evaluated in a retrospective study of 52 patients treated with HDC, in whom echo was performed regularly during a 1-year follow-up (Sandri et al., 2005). NT-proBNP was measured in 52 patients in order to evaluate a possible association with cardiac monitoring findings. Cardiac function was assessed by echo at baseline and at 4 and 12 months after the end of treatment. NT-proBNP samples were measured at baseline, at the end of the infusion, and 12, 24, 36, and 72 h after the end of each CT cycle.

Patients were divided into three groups. Group A, plasma NT-proBNP increased significantly immediately after the end of the infusion, with high concentrations still present after 72 h. Group B, NT-proBNP increased after 12–36 h from the end of treatment but decreased toward baseline after 72 h and group C, NT-proBNP decreased from baseline to 72 h. The mean ratio of peak early to peak late flow velocities (E:A ratio) decreased significantly over time in group A, whereas it remained unchanged in the other two groups. LVEF was significantly decreased only in the A group patients, with the mean value decreased below 50% after 12 months.

Twenty six patients with new acute leukemia, without any previous CT or radiation therapy, were studied in order to compare the changes in biochemical markers with findings on echocardiogram (Horacek et al., 2007). Six months after CT, NT-proBNP concentrations were correlated with systolic and diastolic LV dysfunction on echocardiogram. NT-proBNP’s were elevated in 23/26 patients after the first and last CT with ACs. Six months post completion of CT, 16 patients had persistent high levels and 4 patients had levels above 500 ng/L. Only two patients with NT-proBNP above 500 ng/L, 6 months after treatment, had echo signs of LV dysfunction and clinical symptoms of HF. They were treated with ACEI and diuretics. These two patients had NT-proBNP values markedly elevated already after first and last CT with ANT.

Serum cTNT, BNP levels, and doppler echocardiographic index for the detection and monitoring of AC-induced cardiotoxicity over a period of 1 year in 100 adults undergoing CT were prospectively assessed and compared (Dodos et al., 2008). Patients with no prior use of AC therapy or mediastinal radiotherapy were included. Of note, 18 patients had a history of systemic hypertension which was well controlled under medication. These anti-hypertensive medications may have masked a potential diastolic or systolic cardiac decompensation. Overall, 15 patients developed a cardiac event, defined as a reduction in absolute value >20% in LVEF from baseline or a decline in absolute value >10% in LVEF from baseline and below 55%. Among these patients, seven presented with an additional reduction of the E/A ratio, three with an elevation of cTnT, while none exhibited an alteration of NT-proBNP. No individual with early NT-proBNP increase developed systolic dysfunction, and only four patients presented with a reduction of the E/A ratio during follow-up. All of these patients had normal NT-proBNP levels during further follow-up.

Even though cardiac biomarkers demonstrate some conflicting results in the minimal research we have, they do show some potential promise in their predictive ability of CIC. Their negative predictive value may guide us in risk stratifying our patients and determining which patients need a closer follow-up in the immediate years following CT. Further studies are warranted in order to follow these patients for an extended amount of time post CT and to confirm whether these biomarkers are accurate enough to be able to stratify our patients based on the early results.

#### Echocardiography

As mentioned, patients with cancer are living longer and the acute and late-onset CIC is an ever growing concern. The early detection of systolic or diastolic dysfunction secondary to these agents is an integral part of our management strategies for these patients.

#### Left ventricular ejection fraction

The parameter for monitoring cardiotoxicity has traditionally been the measurement of left ventricular ejection fraction (LVEF). This parameter is most commonly assessed by either echocardiography or multigated acquisition scans (MUGA). Of note, there are no clearly defined guidelines on the monitoring for left ventricular systolic dysfunction for adults undergoing CT, namely with regard to the preferred imaging modality, the frequency of monitoring or what thresholds should be used. The advantages and drawbacks of each of these imaging modalities are stated in Table 2. To further improve the reproducibility and accuracy of LVEF measurement, novel technological advances have been applied in clinical practice. Intravenous administration of contrast agents further enhances endocardial border delineation – through opacification of the left ventricular chamber – and has resulted in improving the intraobserver and inter-observer variability of LVEF measurement (Hoffman et al., 2005). In addition, matrix-array imaging for three-dimensional echocardiography overcomes certain limitations by traditional 2-D echocardiography, such as geometric assumptions, and has shown improved reproducibility and accuracy when compared to 2-D echo using cardiac magnetic resonance imaging as the gold standard (Qi et al., 2007).

**Table 2:** **Imaging modalities in the detection of HF**.

	Echocardiography	MUGA
Advantages	Non-invasive	Lower inter- and observer variability (<5%)
	No radiation	
	Information on valvular function and diastolic dysfunction	Lack of the need for geometric remodeling
Drawbacks	Inter and intra observer variability are 8.8 and 6.8%, respectively (in comparison to MRI)	Exposure to radioactivity

#### Diastolic dysfunction parameters

Relying solely on the measurement of LVEF to detect CT-induced myocardial damage leads us to overlook the subtle alterations of the contractile properties of the myocardium since it is known that LVEF is a late marker of cardiotoxicity. It remains uncertain however whether cardiotoxicity, as defined by the Cardiac Review Committee, can be predicted by any non-invasive index of ventricular function (Stoddard et al., 1992). As in other disease states, alterations in diastole may occur before systolic dysfunction. In fact, several studies have demonstrated that the use of AC alter traditional diastolic parameters measured in echocardiography such as early peak flow velocity to atrial peak flow velocity (E/A), deceleration time (DT), and isovolumetric relaxation time (IVRT; Marchandise et al., 1989; Dorup et al., 2004; Ganame et al., 2007b; Pudil et al., 2008). Other investigators have demonstrated reductions of the tissue-Doppler-derived peak early diastolic velocity of the mitral annulus (E’), a surrogate marker of diastolic dysfunction, in patients receiving AC (Nagy et al., 2006, 2008; Tassan-Mangina et al., 2006; Ho et al., 2010; Sawaya et al., 2011).

Importantly, these decreases in tissue diastolic velocities have not shown any relationship or predictive value for the later development of cardiotoxicity, as defined by the Cardiac Review Committee. In Sawaya et al.’s (2011), the 6-month follow-up failed to demonstrate any predictive value. Whether these global alterations in diastolic function may relate to later onset of cardiotoxicity remains to be studied.

#### Strain and strain rate

As previously stated, because LVEF is a late marker of CIC, novel methods of detecting cardiac damage have been explored and studied. Among those, myocardial strain and strain rate by speckle-tracking imaging have emerged as useful tools in the arsenal of clinical echocardiography.

Strain is defined as a change in length or percent change from the original or unstressed dimension. It reflects the deformation of a myocardial segment and describes the contraction/relaxation pattern of the myocardium. Strain rate is the rate of this deformation and is an index of left ventricular contractility. Three main deformation components can be interrogated: the longitudinal, radial, and circumferential components (5 – Figure 2b of the Mor-Avi et al. guideline document).

Parameters used in 2-D clinical echocardiography, which include fractional shortening (FS) and ejection fraction, evaluate global LV function, and thus, regional myocardial function may be overlooked. This could be important as regional dysfunction may precede global LV dysfunction. Additionally, LVEF and FS values depend largely on preload and afterload. Deformation parameters, such as strain and strain rate, have been demonstrated to be less load dependent and have potential value for the quantification of global and regional systolic and diastolic myocardial function (Ganame et al., 2007b).

One of the first human studies to show decreases in strain from CT was performed on 13 children scheduled to receive AC for various malignancies (Ganame et al., 2007a). Echocardiographic studies were performed before the first dose and <2 h after each of the first three doses of AC to assess its acute cardiac effects. Compared with baseline, a significant decrease in the LV, SF, and LVEF was only evident after the second dose of AC, whereas decreased strain and strain rate decreased significantly after the first dose and remained so after subsequent doses (Ganame et al., 2007a).

In another study by Ganame et al. (2007b), 56 patients with various malignancies (ALL, lymphoma, solid tumors, AML) were studied approximately 5 years after receiving anthracycline therapy to investigate whether changes in strain and strain rate would be detected after CT. Whereas FS and ejection fraction were not different between subjects and controls, peak systolic strain rate and strain values were significantly lower in the anthracycline group. Potential explanations include the fact that cardiac changes may be so localized to only certain myocardial segments, they did not result in changes in global ejection parameters. Moreover, diastolic parameters such as IVRT were altered indicating that small subtle changes may have occurred (Ganame et al., 2007b).

Sawaya et al. (2011) demonstrated that longitudinal strain, in conjunction with cardiac troponin, predicted the development of cardiotoxicity in patients treated with AC and trastuzumab. In this prospective study of 43 women studied prior to, 3 and 6 months after CT, investigators studied whether early echocardiographic measurements of myocardial deformation and biomarkers could predict the development of CIC in patients treated with AC and trastuzumab. Peak systolic radial, longitudinal, and circumferential strain decreased before any decrease in LVEF. Nine patients met the criteria for cardiotoxicity. Decreases in longitudinal strain and radial strain and elevated hsTnI at 3 months were predictive of patients who developed cardiotoxicity at 6 months. The change of LVEF at 3 months was not predictive of later cardiotoxicity. Moreover, patients with both findings had a ninefold increase in the risk of CIC, whereas the absence of either conferred significant protection where only 3% develop CIC. Explanations for these findings have included the fact that CIC may have a regional pattern – the function of some myocardial segments may compensate for other dysfunctional segments leading to a preserved LVEF in the early stages of the cardiotoxicity. Another possible contributing factor explaining for the higher sensitivity of strain compared to the LVEF may be the difference in inter-observer variability. Strain measurement involves the averaging of the automated measurement of multiple segments, whereas LVEF assessment involves a tracing leading to a single measurement.

The same group studied 81 patients over a 1-year follow-up and confirmed that systolic longitudinal myocardial strain and ultrasensitive troponin I measured at the completion of AC therapy were predictive of subsequent cardiotoxicity (Sawaya et al., 2012).

These multiple studies have demonstrated that parameters such as diastolic dysfunction and abnormalities of deformation parameters (strain) precede changes in LVEF and symptomatic HF. It is difficult to state a definite conclusion that alterations to these parameters lead to chronic cardiotoxicity since follow-up times throughout these studies have been relatively short – studies with longer follow-up periods are much needed. However, what we can derive from these studies is that there is a potential opportunity window to introduce cardioprotective medications in order to prevent any long-term sequelae. Joint efforts to further study whether early initiation of cardioprotective medication changes outcome are currently underway.

#### Clinical predictors

Cardiovascular disease had become a significant issue in cancer survivors who have received previous CT, especially ACs. Established modifiable and non-modifiable risk factors such as hypertension, diabetes, and obesity, may predispose individuals to develop CV disease in the context of receiving CT.

In order to investigate the risk factors for the development of late cardiotoxicity following treatment with ACs, a retrospective study analyzed the onset of late cardiac damage by measuring LVEF, and FS in patients treated with doxorubicin for lymphoma (Hequet et al., 2004). Prior to CT, 18% of patients had a diagnosis of hypertension. CV risk factors, such as hypertension, diabetes, hypercholesterolemia, obesity, familial history of cardiac disease, and smoking were analyzed, and only obesity was significantly associated with decreased FS.

Another retrospective cohort study in patients with advanced NHL, who had received at least six cycles of doxorubicin, compared the incidence of CV disease in the EORTC cohort compared with the general population (Moser et al., 2006). The risk of chronic HF was significantly increased in patients treated for aggressive NHL compared to the general population as opposed to a matched risk of coronary artery disease. Age at the start of NHL treatment, pre-existing hypertension, and salvage treatments of any type were risk factors for developing a CV event.

In a large registry, over 40,000 women with breast cancer were analyzed through the Surveillance, Epidemiology, and End Results (SEER) database (Pinder et al., 2007). Coronary artery disease, emphysema, diabetes, hypertension, and peripheral vascular disease were significant predictors of a diagnosis of HF post AC CT. Data from the SEER database were analyzed in order to investigate risk factors of future CV disease in patients with a diagnosis of diffuse large B-cell lymphoma (DLBCL; Hershman et al., 2008). The risk of HF increased in patients with NHL, with the patient’s age and history of coronary heart disease, valvular heart disease, hypertension, diabetes, cigarette smoking, or obesity. Both hypertension and diabetes were associated with an increased prevalence of HF; however in a separate model in this same study, only hypertension was associated with doxorubicin cardiotoxicity.

A retrospective, case-control study, of 63 patients who had undergone hematopoietic stem cell transplant (HCT), evaluated the role of comorbidities in the development of late CV disease (Armenian et al., 2010). Cases were significantly more likely than controls to have been diagnosed with hypertension before HCT and to have a higher BMI at the time of HCT.

There are limited studies investigating the association or the influence of pre-existing CV risk factors on the development of HF post CT. The studies that have been published are mostly in association with ACs and it would be of interest to explore associations with other CT such as TKI’s, which are being administered with increasing frequency. By understanding the impact of CV risk factors on the potential development of CIC, we can further study the impact on prophylactically treating these patients with cardioprotective strategies prior to, throughout, and post CT.

## Conclusion

There are several established pre-existing modifiable and non-modifiable risk factors that predispose individuals to CV disease, some of which include hypertension, diabetes, and obesity. CT agents used in cancer therapy, however, should also be considered themselves as CV “risk factors” when evaluating cancer patients with CV symptoms. Treatment of such patients with established toxicity related to CT remains a challenge for oncologists and cardiologists alike.

In addition, some cancer patients may have no CV symptoms and have pre-clinical findings that may predict the development of symptoms such as HF. Proper screening using novel imaging techniques and serological testing, along with strategies aimed at close clinical monitoring of these patients remains another significant challenge for all specialists involved in the care of cancer patients. Additionally, in the context of multi-modality therapy with chemotherapeutic agents and radiotherapy, clinicians must consider the potential interactions of these treatments. Patients with a history of radiation therapy for malignancies are potentially more susceptible to cardiotoxic effects of standard and novel chemotherapies.

Currently, there are a growing number of centers in North America that are establishing “cardiology-oncology clinics.” Improving communication between specialists in both oncology and CV medicine specialties will not only help advance clinical care, but will also promote clinical research in this rapidly growing field in medicine.

## Conflict of Interest Statement

The authors declare that the research was conducted in the absence of any commercial or financial relationships that could be construed as a potential conflict of interest.
